# Adverse childhood experiences and living in the socially deprived areas in adulthood: a cross-sectional study of the nationwide data in Japan

**DOI:** 10.1186/s12889-023-16557-z

**Published:** 2023-08-24

**Authors:** Natsu Sasaki, Takahiro Tabuchi, Takeo Fujiwara, Daisuke Nishi

**Affiliations:** 1 Department of Mental Health, Graduate School of Medicine, The University of Tokyo, 7-3-1 Hongo, Bunkyo-Ku, Tokyo, 1130033 Japan; 2 Cancer Control Center, Osaka International Cancer Institute, Osaka, Japan; 3 Department of Global Health Promotion, Tokyo Medical and Dental University, Tokyo, Japan

**Keywords:** Maltreatment, Trauma, Socioeconomic status, Equity, Public health

## Abstract

**Objectives:**

This study aimed to examine whether having adverse childhood experiences (ACEs) was associated with living in a deprived area in adulthood.

**Methods:**

The cross-sectional study was conducted by using nation-wide data in 2022 of the Japan COVID-19 and Society Internet Survey (JACSIS). Participants were community dwelling people 18 years or older. ACEs were assessed by Japanese version of 15-items ACE measurement tool (ACE-J). Living condition was measured by Area Deprived Index (ADI) and Densely Inhabited District (DID) based on zip code. Multivariable logistic regression to analyze the associations between ADI and ACE 4 + was conducted, controlling for individual-level factors, such as age, sex, marital status, and education, as an additional analysis.

**Results:**

The total of 27,916 participants were included in the analysis. The prevalence of emotional neglect, childhood poverty, and school bullying were 38.2%, 26.5%, 20.8%, respectively. 75% of the population had one or more ACE(s). The number of ACEs was associated with significantly higher risk of living in deprived area in the adulthood (*p* = 0.001). ACEs were not associated with living in density area. The association between ADI and ACEs 4 + was non-significant after controlling the individual-level factors.

**Conclusion:**

People with higher number of ACEs tend to live in deprived areas in adulthood. Policy makers in highly deprived areas can apply the trauma-informed approach for the community care and support, which is critical to mitigating deficit perspectives and facilitating comprehensive support for those with ACEs.

**Supplementary Information:**

The online version contains supplementary material available at 10.1186/s12889-023-16557-z.

## Introduction

Adverse Childhood Experiences (ACEs), potentially traumatic experiences and events that occur before a child is 18 years old, [1] are critical public health issues. ACEs have negative, lasting effects on mental and physical health and well-being even later in life [1–3]; such as: cardiovascular disease [4], depression [5], diabetes [6], and cancer [7]. The significant impacts of systemic inequality in environment, including the social and economic conditions in which they live, learn, work, and play, may affect the vulnerability for adverse experiences, as social determinants of ACEs [8–10]. Family poverty, low education, less opportunity to work, spirituality, race, parental ACEs, and living conditions are related to increased risk of experiencing ACE(s) [11–15].

Societal factors of living area characteristics, especially living in the socially deprived areas, are highlighted as an important social determinant of health [16]. Neighborhood socioeconomic deprivation in adulthood is associated with high mortality [17–20], lower estimated healthy life expectancy [21], cancer [22], and multimorbidity [23]. Neighborhood deprivation can be measured using various factors such as low income, poor living environment, crime, education, occupation, and housing conditions [24]. Besides, previous studies suggested that neighborhood deprivation and population density might be associated with each other, as deprivation tends to be concentrated in areas with a high population density [22]. The mechanism between area characteristics in adulthood and poor health outcome can be partially explained by health compromising behaviors [25, 26]. ACEs may impact the relationship, since ACEs are one of the major risk factors of diseases as they increase the risky health behaviors [5, 27]. More specifically, we hypothesized that having ACEs may increase the risk of living in deprived areas or density areas, because of 1) poor education, 2) low income, and 3) attachment to the deprived area, as people with higher ACEs are more likely to live in deprived area in childhood [27–29]. A previous study among high school students reported that the level of area deprivation in childhood was associated with increased number of ACEs [14]. In addition, a study reported that the area level prevalence of ACE was independently associated with higher population density [30]. However, the association of having ACEs and living conditions in adulthood is still unknown.

ACEs are negatively associated with future socioeconomic status, such as education, employment, and income potential in adulthood [29], contributing to the cycle of intergenerational effects of ACEs on social deprivation [15, 28, 31–34]. If there is an association of neighborhood deprivation in adulthood with ACEs, it can be useful for considering the appropriate approach based on a deeper understanding of the link between local context and ACEs. Specific intervention considering the deprivation and inequality in living areas could reach overlooked and discriminated communities [35]. As such, the association of area deprivation with ACEs is an important concern in public health.

Furthermore, there is a proposition to broaden the scope of ACEs to extend beyond the parameters of Felitti’s original ACE study [1], which focused on child abuse, neglect, and household dysfunction. The suggested expansion would encompass other forms of ACEs, including peer victimization, which pertains to instances of bullying, exposure to community violence (e.g., living in an unsafe neighborhood), and being a witness to violence [36–39]; which can be frequent and negative impact on future well-being [36–38, 40–42]. Although research about the impacts of expanded ACEs is just beginning, comprehensive exploration of ACEs may be useful in developing area-specific public health measurement.

The authors hypothesized that those with increased number of ACEs were likely to live in urban and deprived area in adulthood due to the intergenerational effects of ACEs on social deprivation. The aim of this study is to examine between the association of the number of expanded ACEs and current living area characteristics measured by neighborhood deprivation and population density by using data of the nation-wide large study. Understanding the connection between neighborhood deprivation and ACEs in adulthood can provide valuable insights for determining an appropriate approach that takes into account the local context and disadvantages of people living there.

## Method

### Study design

This study used data from the Japan COVID-19 and Society Internet Survey (JACSIS). JACSIS is a nation-wide online cohort study in Japan, conducted since August 2020 to the present. Participants were community dwelling people aged 16–79 years. The baseline sample of JACSIS was collected in 2020 (*n* = 28,000). A follow-up survey was conducted in 2022 for the participants in 2020 or 2021, and new participants were invited to join the 2022 survey, creating a total of *N* = 32,000 people participated the 2022 survey. In this study, a cross-sectional design was applied using the JACSIS 2022 data.

### Recruitment

The study utilized email messages to request survey participation from a research panel consisting of individuals aged 15 to 79 years who were registered with Rakuten Insight, Inc. The company’s registered panel consisted of over 2.2 million individuals with diverse sociodemographic backgrounds, in Japan. Potential participants were selected using a simple random sampling method based on sex, age, and prefecture category in accordance with the official Japanese demographic composition as of October 1, 2019. Those who agreed to participate in the survey were given access to a designated website. Participants were allowed to skip any questions or discontinue the survey at any point. Priority was given to participants who had completed the survey in 2020 or 2021 during the 2022 data collection, but new participants were also invited until the final sample size was reached (*N* = 32,000).

### Management of data quality

To ensure the validity of the data, respondents who exhibited discrepancies or provided artificial/unnatural responses were removed from the study. Specifically, three question items were used to identify such responses: “Please choose the second from the bottom,” “choosing positive in all of a set of questions for using drugs,” and “choosing positive in all of a set of questions for having chronic diseases.” Respondents who were found to have provided such responses (*n* = 3320) were excluded from the study.

### Participants

The study sample included community dwelling people in Japan over 18 years old and without missing data. Following these criteria, participants aged under 18 (*n* = 13) and participants with missing data of area deprivation index (*n* = 700) were excluded.

### Measurement variables

#### Adverse Childhood Experiences (ACEs)

The Adverse Childhood Experiences Japanese version (ACE-J) is a questionnaire that assesses individuals’ exposure to ACEs in Japan [36]. The items were limited to one for each category of adversities, except parental loss. Parental loss was assessed as either parental death and divorce or separation. Following the expanded concept of ACEs, childhood poverty, bullying, hospitalization due to chronic disease, and exposure to life-threatening natural disasters were included. The ACE-J consisted of 15 items in total. Participants were asked whether they had following experiences under 18 years old. The response options were “Yes” or “No.” One item related to emotional neglect was reversed question; “I felt I was loved by my parents.” After reversing the score of this item, the summed score of the ACEs were used as the number of ACEs experienced.

#### Living area characteristics

The Area Deprivation Index (ADI) and Densely Inhabited District (DID) were used to assess living area characteristics. Both indices were created at a zip code level in Japan, with a total of 113,107 zip codes included in the analysis. Each zip code included approximately 1,100 residents. The zip code was directly obtained in the online questionnaire by asking participants about the zip code of their current living area.

##### Area Deprived Index (ADI)

In this study, the Area Deprivation Index (ADI) was utilized as a measure of neighborhood deprivation. The data used to construct the ADI was obtained from the 2010 Population Census of Japan, and the specific methodology used to calculate the ADI has been previously described [17]. The ADI is a composite indicator that considers several poverty-related census variables, including the unemployment rate, proportion of elderly couple households, elderly single-occupier households, single mother households, rented houses, sales and service workers, agricultural workers, and blue-collar workers. A higher ADI score is indicative of greater neighborhood deprivation. In this study, the ADI was divided into four categories by using quartile to facilitate analysis, following a previous study [43].

##### Densely Inhabited District (DID)

The urbanization level of the study areas was measured using the Densely Inhabited District (DID) data obtained from the 2015 Population Census of Japan. The DID score is an indicator of the level of population density in a given area, with a higher score indicating a greater level of urbanization. To facilitate analysis, the DID score was dichotomized into high and low categories. The data source for this information is publicly available [44]. DIDs that lack a zip code centroid were deemed non-DID, indicating a rural area, and were allocated to the urbanization level with the lowest category. DIDs that possess a zip code centroid, on the other hand, were classified as DID, denoting an urbanized area, and were arranged into tertiles based on population density. These tertiles correspond to the second, third, and highest categories of urbanization level. This classification followed the previous study, which examine the association of ADI and DID and mental health [43].

### Sociodemographic characteristics

Age, sex, educational attainment (less than high school, vocational/college, undergraduate, graduate over), and marital status (married, single/divorced), household income (< 3, 3 -5, 5–8, 8 – 10, over 10 million yen, no response/unknown), and working status (paid work, no paid work, students) were measured as demographic variables.

### Statistical analysis

The descriptive statistics including the prevalence of ACEs were estimated by using the inversed propensity score as the sampling weight to address a potential sampling bias due to online survey. The propensity score was calculated based on a demographic distribution of a national paper-based survey, the Comprehensive Survey of Living Conditions of People on Health and Welfare (CSLCPHW), uisng sex and age group stratifications (sex × age groups = 14 strata). The associations of ACEs with ADI and DID were examined using one-way ANOVA with sampling weight. Considering neighborhood deprivation as a reflection of people’s disadvantaged sociodemographic status, the crude model was considered the primary analysis. To explore the effects of individual-level factors, the authors employed multivariable logistic regression to analyze the associations between the number of ACEs and living in highly deprived area (top category by using quartile), controlling for age and sex (Model 1), marital status, education, and sample weighting scores (Model 2), as an additional analysis. The same analysis was conducted inversely using the outcome as ACE 4 + . Another logistic regression was conducted to examine the associations of ADI with ACEs. As no relevant area-based information was available, multi-level analysis was not conducted in the current study.

## Results

Table 1 shows the participants demographics of this study. A total of 27,916 participants were included in the analysis. Elderly people over 60 years old (30%), married (62%), less than high school educational attainment (56%), and having paid work (65%) were majority of the participants.

**Table 1:** Weighted participant demographics (*N* = 27916)

	N (Percentage)	Mean (SD)
Sex		
Male	13599 (48.7)	
Female	14317 (51.3)	
Age		48.2 (17.3)
18—19 years old	203 (0.7)	
20—29 years old	4423 (15.8)	
30—39 years old	6154 (22.0)	
40—49 years old	4634 (16.6)	
50—59 years old	4041 (14.5)	
Over 60 years old	8460 (30.2)	
Marital status		
Married	17377 (62.2)	
Single/divorced	10539 (37.8)	
Educational attainment		
Less than high school	15528 (55.6)	
Vocational/College	5336 (19.1)	
Undergraduate	6419 (23.0)	
Graduate over	632 (2.3)	
Household income		
< 3 million yen	5160 (18.5)	
3—5 million yen	5831 (20.9)	
5—8 million yen	6089 (21.8)	
8—10 million yen	2206 (7.9)	
Over 10 million yen	2223 (8.0)	
No response/unknown	6407 (23.0)	
Work		
Paid work	18015 (64.5)	
No paid work	9031 (32.4)	
Students	869 (3.1)	

*SD* Standard deviation

Table 2 shows the prevalence of ACEs measured by the ACE-J. The prevalence of emotional neglect (38%), childhood poverty (27%), and school bullying (21%) were high. Mean of the ACEs was 1.75 (SD = 1.94). The finding shows that 75% of the population had one or more ACEs. The prevalence of people with ACE 4 + was 14.6%.

**Table 2:** Weighted prevalence of Adverse Childhood Experiences (ACEs) measured by ACE-J in community dwelling Japanese population (*N* = 27916)

Adverse Childhood Experiences	Percentage Mean (SD) [95%CI]
Parental loss	
Death	10.1
Divorce	10.7
Mental illness in the household	4.3
Substance abuse in the household	6.8
Mother treated violently	8.7
Physical abuse	3.8
Physical neglect	3.2
Emotional abuse	12.8
Emotional neglect^a^	38.2
Childhood poverty	26.5
Overcontrol	15.4
School bullying	20.8
Sexual abuse	4.4
Hospitalization due to chronic disease	4.9
Exposure to life-threatening natural disaster	3.4
The number of ACEs	1.75 (1.94) [0 – 15]
ACE 0	25.4
ACE 1	35.5
ACE 2	15.7
ACE 3	8.8
ACE 4 +	14.6

*SD* Standard deviation, *CI* Confidential interval

^a^Reverse scored item

Table 3 shows the association of the number of ACEs with the association of living area characteristics measured by ADI and DID. The analysis presented that the proportion of those living in highly deprived are was significantly upgraded as the number of ACEs was increased (*p* = 0.001). However, the analysis did not show a significant association with DID.

**Table 3:** Weighted associations of the number of ACEs with the highly deprived area (top category by using quartile) measured by Area Deprived Index (ADI) and highly urbanized area (top category by using quartile) measured by Densely Inhabited District (DID) in Japan (*N* = 27916)

		Living in highly deprived area	Living in highly urbanized area
	N	Percentage (%)	Percentage (%)
The number of ACEs			
ACE 0	7096	26.6	20.1
ACE 1	9897	28.0	20.1
ACE 2	4375	28.1	20.3
ACE 3	2459	30.5	20.2
ACE 4 +	4089	29.4	19.4
Difference^a^		χ2 = 17.81, *p* = 0.001	χ2 = 1.25, *p* = 0.870

*SD* Standard deviation, *ACE* Adverse childhood experience, *ACE 4* + More than four adverse childhood experiences

^a^Group differences were tested by chi-square test

The result of the multivariable logistic regression for living in highly deprived area as an outcome is shown in Table 4. In the crude model, ACEs (4 +) was significantly associated with living in highly deprived area in the adulthood, compared to those without ACE (OR = 1.21 [1.11 – 1.32]). In the adjusted model including weighting score as covariates (Model 2), the individual factors (i.e., single, less educated) were significantly associated with living in highly deprived area, and the number of ACEs did not maintain its significance.

**Table 4:** Logistic regression to examine the associations of ACEs with the living in highly deprived area (top category by using quartile) measured by Area Deprived Index (ADI) in Japan, adjusted covariates and sample weighting score (*N* = 27921)

	Crude		Adjusted (Model 1)^a^		Adjusted (Model 2)^b^	
	OR	95% CI	aOR	95% CI	aOR	95% CI
The number of ACEs (ref.0)						
ACE 1	1.07	1.00 – 1.15	1.08	1.01 – 1.16	1.03	0.96 – 1.11
ACE 2	1.11	1.02 – 1.21	1.11	1.02 – 1.21	1.02	0.94—1.12
ACE 3	1.15	1.03 – 1.28	1.15	1.04 – 1.28	1.05	0.94 – 1.17
ACE 4 +	1.21	1.11 – 1.32	1.22	1.12 – 1.34	1.08	0.99 – 1.18
Age			1.00	1.00—1.01	1.00	1.00 – 1.01
Female (ref: male)			1.00	0.95 – 1.06	0.92	0.87 – 0.98
Single (ref: married)					1.25	1.18 – 1.33
Weighting score					1.02	1.00 – 1.04
Education (ref: less than high school)						
Vocational/College					0.87	0.80 – 0.94
Undergraduate					0.64	0.59 – 0.89
Graduate over					0.50	0.43 – 0.58

The total number of analyzed participants was different from the main tables because this analysis was not weighted but the weighting score was added as a covariate in model 2

*CI* Confidential intervals, *OR* Odds ratio

^a^Adjusted by age and sex

^b^Adjusted by age, sex, education, and sample weighting score

Supplementary table 1 shows the association of living area characteristics measured by ADI and DID with the number of ACEs and ACE 4 + . The number of ACEs and the prevalence of ACE 4 + were significantly increased as the neighborhood deprivation level (ADI) was upgraded (*p* < 0.001). The analysis of DID showed the inconsistent significance in the number of ACEs and ACE 4 + .

The result of the multivariable logistic regression for the association of neighborhood deprivation with ACEs is shown in Supplementary Table 2. In the crude model, living in the very high deprived area was significantly associated with high ACEs (4 +), compared to living in very low deprived area (odds ratio 1.21 [95% confidential intervals: 1.10 – 1.33]). In the adjusted model including weighting score as covariates, the individual factors (i.e., female, single, less educated) were significantly associated with high ACEs (4 +), and ADI did not maintain its significance.

Supplementary Table 3 shows the association of each ACE with living in highly deprived area in adulthood. In the adjusted model including weighting score as covariates, exposure to life-threatening natural disaster showed the most significant association with living in highly deprived area (aOR = 1.21 [1.05—1.40]).

## Discussion

The findings showed that people with a higher number of ACEs live in more deprived areas. The area characteristics of population density was not associated with ACEs. While the individual factors more influenced ACEs than the current living conditions, this finding is meaningful to reveal the target in public health, which implements supports for people with great number of ACEs.

The increased number of ACEs was observed in people living in deprived areas. This result was in line with the previous study indicating that high school students living in deprived area were more likely to have increased ACEs [14]. The possible mechanisms were: (i) intergenerational effect of individual deprivation due to limited family resources in childhood, and (ii) social disadvantages which caused an increased number of ACEs, forcing them to live in deprived area in adulthood. For the mechanism (i), low socioeconomic status can be transmitted to next generations [33, 34]. The association which this study found was thus the proximal of the association of local context in childhood with ACEs, as the “ACE Pyramid” suggested that social conditions/local context can produce vulnerability to experience ACEs [9]. Children in low socioeconomic families tend to have many ACEs due to increased parental stress and limited capacity [15, 32] as well as limited access to resources to avoid ACEs [8]. For mechanism (ii), even if those living in deprived area in adulthood did not live in deprived area in childhood, ACEs may push people to live in deprived area in adulthood, because ACEs negatively affect socioeconomical positions [31]. Nonetheless, a deprived area is an important target to be implemented the interventions considering ACEs.

There was no significant association of population density with ACEs, against a previous study, which reported that the area level prevalence of ACEs was associated with the population density in England [30]. Population density has a consistent economic effect, but it is difficult to claim the association with individual adversities. Urban/rural distinction may not necessarily reflect social resources and adversities of those living there. The previous study also stated that additional factors to explain the associations more directly, such as economic history, the presence of local criminal activities and availability and quality of specific public services, should be considered [30]. A such, support for those with ACEs can be essential regardless of the level of urbanity.

The association of area deprivation with ACEs was not significant after controlling for individual socioeconomic factors. This was consistent with a previous study which showed that people with great number of ACEs are likely to have low socioeconomic status in adulthood [29]. The effect of ACEs on living area selection in adulthood may be more indirect, occurring through the collective influence of an individual’s factors. Otherwise, it was possible that conceptual overlap between individual deprivation and area level of deprivation weakened the associations of area characteristics with ACEs. However, the authors believe that the findings about the crude association of area characteristics with ACEs are more important than adjusted model. The present findings in this study suggested the potential needs to implement specific intervention considering ACEs, such as trauma-informed approach [45], in more deprived areas. Although the interventions are ideally needed to be implemented in all area, considering that people with ACEs can be in all area, this study showed the priority and urgent necessity of it in more deprived areas. The indirect path with the relationship between regional characteristics and ACEs through individual socioeconomic factors may be stronger than the direct path of it. But the present finding still showed the importance of focusing on the regional characteristics, considering the difficulty of implementing the public health measurement, which targets individuals with specific socioeconomic status.

This study found that 75% of the population had one or more ACEs, and 14.6% of adults have experienced four or more ACEs by using 15-items of ACE-J scale. In expanded ACE study (The Philadelphia Urban ACE Study), the prevalence was reported 83.2% had at least one ACEs and 37.3% experienced four or more ACEs, measured by 14 items with additional stresses including bullying [39]. However, Japan has relatively fewer ACEs compared to other countries. A previous study in Japan reported less prevalence of those with one or more ACEs (32%) [46], than the prevalence of the original ACE study, which reported 63.9% had one or more ACEs, and 12.5% had four or more ACEs, measured by 10 items of ACEs in U.S. [9]. The prevalence of ACEs reported in this study was in line with the current trend of studies. Regarding school bullying, 21% of participants had experienced in this study. This was consistent with the previous report that 22% of children reported being victims of any type of bullying a few times a month or more in Japan, which is higher than the Organization for Economic Co-operation and Development (OECD) average of 19% [47]. The prevalence of ACEs found in this study can be considered reasonable.

### Limitation

This study has several limitations. The cross-sectional nature of the study design prohibited the authors to consider the causal relationship. However, the authors presume that the number of ACEs under 18 years old cannot be changed through the life course, and cross-sectional study is enough to grasp the epidemiological information about whether people with great number of ACEs are likely to live in a deprived area. Additionally, while area characteristics in childhood can be a critical confounder, the consistency of area characteristics in childhood and in adulthood was unknown, because of unavailability of information about where they lived in childhood. Self-repointing questionnaire lead potential measurement bias. For example, the measurement of ACEs may be affected by recall bias. The ADI and DID was created based on the previous census data; it was possible to be different from the current situation. The ADI use has a limitation: it cannot reflect details of the structural characteristics, especially in urban areas with expensive housing closely adjacent to poor housing. Another limitation is that the online survey could lead a sampling bias. In spite of our best effort to reduce the bias by utilizing the weighted analysis, people with high literacy of internet technology may tend to participate in the study. Generalizability is thus limited. In addition, the broad age range of study participants in this study may impact individual socioeconomic factors, possibly leading to an underestimation of the associations between ACEs and ADI. The current analysis did not adequately examine the associations between individual and regional factors and between individual factors and ACEs. Due to conceptual overlap, we did not select income or work status as a covariate because they are already included in the ADI concept. However, the current analysis did not adequately account for factors that lie between regional factors and ACEs, and we were unable to demonstrate a mechanism. Future studies should examine further complex relationships among individual characteristics, regional factors, and ACEs.

## Conclusion

This study examined the association of the number of expanded ACEs with the currently living area characteristics measured by neighborhood deprivation and population density by using data of the Japanese nationwide study. The findings demonstrated that the number of ACEs was associated with the level of neighborhood deprivation, while individual socioeconomic factor more affected than area characteristics. Policy makers in highly deprived area can apply the trauma-informed approach for the community care and support, which is critical to mitigating deficit perspectives and facilitating comprehensive support for those with ACEs. Besides, preventive and resilience-enhancing supports that continue from infancy to adolescence and beyond are needed for children and families, and the need to increase the strength of the community as a whole can be considered to mitigate the negative impact of ACEs.

### Supplementary Information

**Additional file 1: Supplementary Table 1.** Associations of the current living area characteristics measured by Area Deprived Index (ADI) and Densely Inhabited District (DID) with ACEs in Japan (*N*=27916). **Supplementary Table 2.** Logistic regression to examine the associations of the current living area characteristics measured by Area Deprived Index (ADI) with ACEs>4 in Japan, adjusted covariates and sample weighting score (*N*=27921). **Supplementary Table 3.** Logistic regression to examine the associations of each ACE with the living in highly deprived area (top category by using quartile) measured by Area Deprived Index (ADI) in Japan, adjusted covariates and sample weighting score (*N*=27921).

## Data Availability

The data used in this study are not available in a public repository because they contain personally identifiable or potentially sensitive patient information. Based on the regulations for ethical guidelines in Japan, the Research Ethics Committee of the Osaka International Cancer Institute has imposed restrictions on the dissemination of the data collected in this study. All data enquiries should be addressed to the person responsible for data management, Dr. Takahiro Tabuchi, at the following e-mail address: tabuchitak@gmail.com.
