# Supplementary material for: Adverse childhood experiences and living in the socially deprived areas in adulthood: a cross-sectional study of the nationwide data in Japan
Source: BMC Public Health. 2023 Aug 24;23:1616. doi: 10.1186/s12889-023-16557-z (PMC10463887; doi:10.1186/s12889-023-16557-z)
Supplement: Supplementary file 1 — Additional file 1: Supplementary Table 1. Associations of the current living area characteristics measured by Area Deprived Index (ADI) and Densely Inhabited District (DID) with ACEs in Japan (N=27916). Supplementary Table 2. Logistic regression to examine the associations of the current living area characteristics measured by Area Deprived Index (ADI) with ACEs>4 in Japan, adjusted covariates and sample weighting score (N=27921). Supplementary Table 3. Logistic regression to examine the associations of each ACE with the living in highly deprived area (top category by using quartile) measured by Area Deprived Index (ADI) in Japan, adjusted covariates and sample weighting score (N=27921). [file 12889_2023_16557_MOESM1_ESM.docx]

**Supplementary Table 1.** **Associations of the current living area characteristics measured by Area Deprived Index (ADI) and Densely Inhabited District (DID) with ACEs in Japan (****N=27916).**

|  |  | Total number of ACEs | Prevalence of ACE 4+ |
| --- | --- | --- | --- |
|  | N | Mean (SD) | Percentage |
| ADI |  |  |  |
| Very low deprivation | 5411 | 1.65 (1.85) | 13.5 |
| Low deprivation | 6971 | 1.75 (1.94) | 14.2 |
| High deprivation | 7686 | 1.74 (1.94) | 15.2 |
| Very high deprivation | 7848 | 1.82 (2.00) | 15.3 |
| difference ^a^ |  | F=8.26, p<0.001 | χ2=12.15, p=0.007 |
| DID |  |  |  |
| No DID | 9715 | 1.79 (1.96) | 14.7 |
| Low urbanization | 4308 | 1.68 (1.93) | 14.4 |
| Middle urbanization | 8301 | 1.76 (1.96) | 15.0 |
| High urbanization | 5591 | 1.71 (1.86) | 14.2 |
| difference ^a^ |  | F=4.04, p=0.007 | χ2=1.99, p=0.574 |

SD: standard deviation. ACE: adverse childhood experience. ACE 4+: more than four adverse childhood experiences.

^a^ Group differences were tested by one-way ANOVA for the total number of ACEs and by chi-square test for the prevalence of ACE 4+.

**Supplementary Table 2. Logistic regression to examine the associations of the current living area characteristics measured by Area Deprived Index (ADI) with ACEs>4 in Japan, adjusted covariates and sample weighting score (N=27921)**

|  | Crude |  | Adjusted (Model 1) ^(a^ | | Adjusted (Model 2) ^(b^ | |
| --- | --- | --- | --- | --- | --- | --- |
|  | OR | 95% CI | aOR | 95% CI | aOR | 95% CI |
| ADI (ref: very low) |  |  |  |  |  |  |
| Low | 1.03 | 0.93 – 1.14 | 1.04 | 0.94 – 1.15 | 0.97 | 0.87 – 1.07 |
| High | 1.15 | 1.04 – 1.27 | 1.15 | 1.04 – 1.27 | 1.06 | 0.96 – 1.17 |
| Very high | 1.21 | 1.10 – 1.33 | 1.22 | 1.11 – 1.35 | 1.07 | 0.97 – 1.18 |
| Age |  |  | 0.99 | 0.99 – 1.00 | 0.99 | 0.99 – 0.99 |
| Female (ref: male) |  |  | 1.46 | 1.36 – 1.57 | 1.36 | 1.26 – 1.46 |
| Single (ref: married) |  |  |  |  | 1.38 | 1.28 – 1.49 |
| Weighting score |  |  |  |  | 0.97 | 0.94 – 0.99 |
| Education (ref: less than high school) |  |  |  |  |  |  |
| Vocational/College |  |  |  |  | 0.68 | 0.61 – 0.75 |
| Undergraduate |  |  |  |  | 0.45 | 0.41 – 0.50 |
| Graduate over |  |  |  |  | 0.54 | 0.45 – 0.65 |

CI: confidential intervals. OR: odds ratio.

a) Adjusted by age and sex.

b) Adjusted by age, sex, education, and sample weighting score.

*Note*. The total number of analyzed participants was different from the main tables because this analysis was not weighted but the weighting score was added as a covariate in model 2.

**Supplementary Table 3** **Logistic regression to examine the associations of each ACE with the living in highly deprived area (top category by using quartile) measured by Area Deprived Index (ADI) in Japan, adjusted covariates and sample weighting score (N=27921)**

|  | Crude |  | Adjusted (Model 1) ^(a^ | | Adjusted (Model 2) ^(b^ | |
| --- | --- | --- | --- | --- | --- | --- |
|  | OR | 95% CI | aOR | 95% CI | aOR | 95% CI |
| Parental loss |  |  |  |  |  |  |
| death | 1.07 | 0.98 - 1.17 | 1.03 | 0.94 - 1.13 | 0.98 | 0.90 - 1.08 |
| divorce | 1.19 | 1.08 - 1.30 | 1.22 | 1.11 - 1.33 | 1.11 | 1.01 - 1.22 |
| Mental illness in the household | 0.97 | 0.84 - 1.11 | 1.00 | 0.87 - 1.15 | 0.96 | 0.84 - 1.10 |
| Substance abuse in the household | 1.20 | 1.08 - 1.34 | 1.21 | 1.08 - 1.35 | 1.12 | 1.00 - 1.25 |
| Mother treated violently | 1.12 | 1.02 - 1.23 | 1.11 | 1.01 - 1.22 | 1.05 | 0.96 - 1.16 |
| Physical abuse | 1.12 | 0.97 - 1.29 | 1.14 | 0.99 - 1.31 | 1.07 | 0.93 - 1.24 |
| Physical neglect | 1.09 | 0.93 - 1.28 | 1.10 | 0.93 - 1.29 | 1.02 | 0.86 - 1.20 |
| Emotional abuse | 1.04 | 0.96 - 1.13 | 1.06 | 0.97 - 1.15 | 1.02 | 0.94 - 1.10 |
| Emotional neglect a | 1.01 | 0.95 - 1.07 | 1.04 | 0.98 - 1.10 | 0.98 | 0.93 - 1.04 |
| Childhood poverty | 1.24 | 1.16 - 1.32 | 1.22 | 1.14 - 1.30 | 1.11 | 1.04 - 1.18 |
| Overcontrol | 1.05 | 0.98 - 1.14 | 1.06 | 0.98 - 1.14 | 1.01 | 0.93 - 1.09 |
| School bullying | 1.04 | 0.97 - 1.11 | 1.06 | 0.99 - 1.14 | 1.03 | 0.96 - 1.10 |
| Sexual abuse | 1.06 | 0.93 - 1.21 | 1.06 | 0.93 - 1.21 | 1.02 | 0.89 - 1.17 |
| Hospitalization due to chronic disease | 0.95 | 0.83 - 1.09 | 0.94 | 0.82 - 1.07 | 0.90 | 0.79 - 1.03 |
| Exposure to life-threatening natural disaster | 1.23 | 1.07 - 1.42 | 1.23 | 1.06 - 1.42 | 1.21 | 1.05 - 1.40 |

CI: confidential intervals. OR: odds ratio.

a) Adjusted by age and sex.

b) Adjusted by age, sex, education, and sample weighting score.

*Note*. The total number of analyzed participants was different from the main tables because this analysis was not weighted but the weighting score was added as a covariate in model 2.
